# Assessing upper-extremity motion: An innovative method to quantify functional capacity in patients with chronic obstructive pulmonary disease

**DOI:** 10.1371/journal.pone.0172766

**Published:** 2017-02-24

**Authors:** Nima Toosizadeh, Cristine Berry, Christian Bime, Bijan Najafi, Monica Kraft, Jane Mohler

**Affiliations:** 1 Arizona Center on Aging, University of Arizona, Department of Medicine, Tucson, Arizona, United States of America; 2 Interdisciplinary Consortium on Advanced Motion Performance (iCAMP), Department of Surgery, University of Arizona, Tucson, Arizona, United States of America; 3 Pulmonary, Allergy, Critical Care, & Sleep Medicine, Department of Medicine, University of Arizona, Tucson, Arizona, United States of America; 4 Division of Vascular Surgery and Endovascular Therapy, Michael E. DeBakey Department of Surgery, Baylor College of Medicine, Houston, Texas, United States of America; Lee Kong Chian School of Medicine, SINGAPORE

## Abstract

**Background:**

Assessment of functional capacity is important in directing chronic obstructive pulmonary disease (COPD) care (e.g., rehabilitation and discharge readiness), and in predicting outcomes (e.g., exacerbation, hospitalization, and mortality). The 6-minute walk distance (6MWD) test for functional capacity assessment, may be time-consuming and burdensome.

**Objective:**

The purpose of the current study was to evaluate an upper-extremity function (UEF) test for assessing functional capacity in older adults with COPD.

**Methods:**

In this cross-sectional study, 49 older adults (≥55 years) with diagnosed COPD were recruited, and pulmonary function measures and 6MWD were obtained. Participants wore wireless sensors on forearm and upper-arm and performed rapid elbow flexion for 20 seconds (the UEF test). Slowness was assessed by measuring elbow speed, and acceleration and weakness (muscle strength) were assessed by measuring power of movement and elbow moment.

**Results:**

Speed, power, and moment UEF parameters were independently associated with 6MWD, when controlling for age, gender, and body mass index (BMI) (*r* = 0.78, *p* < .001). Elbow moment showed significant Pearson correlations with all pulmonary function measures and maximal inspiratory/expiratory pressure measures (*r* = 0.35–0.69, *p*<0.02).

**Conclusions:**

Results show promise of a quick upper-extremity measure of functional capacity in patients with COPD, and as an outcome measure in clinical COPD trials.

## Introduction

Chronic obstructive pulmonary disease (COPD) is a complex disease, resulting in substantial burden to patients’ and health care systems, and is the third leading cause of death in the United States among adults [1,2]. Furthermore, COPD is associated with impairment of physical functioning, resulting in limitations in ability to perform activities of daily living [3]. The diagnosis and severity of COPD are determined by pulmonary function measurement using spirometry, with low pulmonary function (measured by forced expiratory volume in one second—FEV_1_) predicting worse health outcomes [4]. While spirometry plays a central role in diagnosis and staging, it sometimes fails to reliably reflect COPD symptomology or the burden on patients’ functioning [5]. Assessment of functional capacity, in addition to pulmonary function measures can be a practical approach in the prediction of COPD health outcomes. The 6-minute walk distance (6MWD) test was developed in 1963 by Balke to evaluate functional capacity [6]. Later on, 6MWD was tested among COPD patients and showed moderate to strong correlations (*r* = 0.56 to *r* = 0.88) with the peak VO_2_ obtained by maximal exercise testing [7]. More recently, the 6MWD test has been commonly used to assess functional capacity in COPD patients and has been shown to predict mortality better than traditional pulmonary function measures [8,9]. Although 6MWD has good reliability and validity as a measure of functional capacity in COPD [9,10], performing the test is time-consuming (six minutes testing plus an additional ~1 minute for preparation [11]) and may be burdensome for some patients, especially older patients with mobility impairments, bedbound inpatient population, and those on wheelchairs. Other contraindications for the 6MWT include: unstable angina month and myocardial infarction during the previous month, relative contraindications include a resting heart rate of more than 120, a systolic blood pressure of more than 180 mm Hg, and a diastolic blood pressure of more than 100 mm Hg [12,13]. There are also a few other factors that may influence the distance a patient can walk safely including significant medical co-morbidities such as frailty, postural instability, musculoskeletal limitations, high fall risk, significant peripheral neuropathy and advanced dementia [12,13]. 6MWD results can be influenced by the height and weight of the patient, as well as changes in track layout and length and use of wheeled walkers [14,15]. Further, the 6MWD may be impractical in understaffed or small clinical settings. For these reasons, an alternative objective and simple approach for assessing functional capacity in COPD may prove beneficial.

We have previously developed and validated an upper-extremity function (UEF) test to assess slowness, weakness, exhaustion, and flexibility [16,17]. The objective UEF assessment method integrates low cost motion sensors, and the assessment (including post-processing) is easily performed with the instruction of a medical assistant in less than one minute. Additionally, in the previous study we determined strong correlations between upper-extremity motion and gait speed [17]. In COPD, gait speed has been demonstrated to be accurate in identifying clinically relevant benchmarks of the 6MWD test [18]. In other work, upper-extremity muscle strength has shown to be less in COPD patients compared to healthy participants [19]. As such, the purpose of the current study was to evaluate the UEF test as an assessment for functional capacity in older adults with COPD. We specifically investigated whether UEF parameters correlate with 6MWD and pulmonary function measures. Since age-associated loss of muscle strength (dynapenia) and slow gait speed are common in older COPD patients [20,21], we hypothesized that UEF parameters predictive of weakness and slowness within the upper-extremity motion would significantly correlate with 6MWD and pulmonary function measures.

## Materials and methods

### Participants

Aging adults (≥55 years) were recruited from Banner University Medical Center Tucson pulmonary clinics and pulmonary function laboratory from September 2014 to February 2015. COPD diagnosis was determined based on physician assessment and confirmed by spirometry (post-bronchodilator FEV_1_/FVC <0.7; FVC = forced vital capacity). Participants were excluded if they had any clinically severe neurologic or neuromuscular condition that could, in the judgment of the investigators, interfere with the ability to participate in the study (including stroke or Parkinson’s disease). The study was approved by the University of Arizona Institutional Review Board. Written informed consent according to the principles expressed in the Declaration of Helsinki [22] was obtained from all subjects before participation.

### Clinical measurements and questionnaires

Pulmonary function measures included: FEV_1_; FVC; peak expiratory flow rate (PEFR); maximal inspiratory pressure (MIP); and maximal expiratory pressure (MEP). All pulmonary function measures were obtained according to American Thoracic Society recommendations [23]. COPD GOLD stages (0–4: mild to severe) were defined for participants similar to previous work [24]. Pulmonary function measures were performed using a spirometer (Vyntus SPIRO; San Diego, CA). In addition, the COPD assessment test (CAT) [25] was completed by each participant to assess COPD symptoms.

### UEF and 6MWD tests

**Fig 1:**
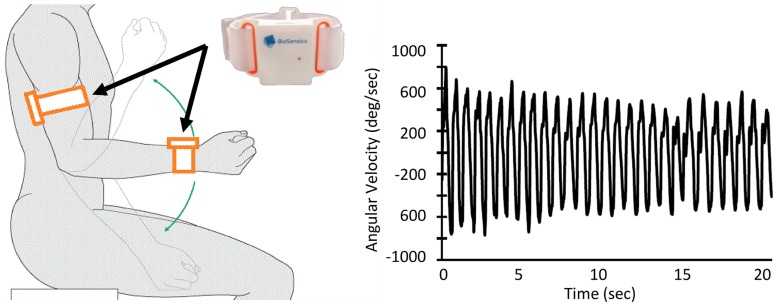
UEF experimental setup and sensors output (i.e., elbow angular velocity).

Each participant performed a ~20-second trial of elbow flexion, within which they repetitively flexed and extended their dominant elbow to full flexion and extension as quickly as possible in seated position, while wearing the UEF system (Fig 1). This task involves elbow flexion mainly using biceps branchii, deltoidues p.clavicularis, and branchioradialis muscles and elbow extension dominantly using triceps branchii, deltoidues p.spinata, and amconeus muscles. Before the actual test, participants performed one practice trial on their non-dominant side to become familiar with the protocol. The protocol was explained to participants by a trained para-medical personnel, and they were encouraged only once, before elbow flexion, to do the task as fast as possible (participants were not further encouraged during the task). A tri-axial wearable gyroscope sensor (sample frequency 100Hz, BioSensics LLC, Brookline, MA), was attached to the upper-arm near the biceps and one to the wrist using a band attached with Velcro, to estimate three-dimensional angular velocity of the upper-arm and forearm segments, and ultimately elbow flexion.

Several outcome measures representing kinematics and kinetics of elbow flexion were derived using angular velocity and anthropometric data (i.e., participants’ stature and body mass). Outcome measures included: 1) speed; 2) range of motion; 3) power; 4) rise time; 5) moment; 6) speed variability; 7) speed reduction; and 8) flexion number (see Table 1 for definitions). These parameters were defined to quantify “slowness”, “weakness”, “exhaustion”, and “flexibility”. Slowness was assessed by measuring speed, rise time, and flexion number; acceleration and weakness was assessed by measuring power and moment; exhaustion was assessed by speed variability and speed reduction. (Readers are referred to [17] for more details regarding validation of UEF using a reference motion capture system, and detailed description of parameter calculations.)

**Table 1:** UEF parameter definitions. See (15) for more details.

Parameter	Definition
Speed	Mean value (within 20-second elbow flexion/extension) of elbow angular velocity range (maximum minus minimum speed)
Range of motion	Mean value of elbow flexion range
Power	Mean value of product of the angular acceleration range and the range of angular velocity
Rise time	Mean value of time required to reach the maximum angular velocity
Moment	Mean value of maximum moments on elbow within each flexion/extension estimated from moment of inertia of forearm and hand, and elbow motion
Speed variability	Coefficient of variation (standard deviation divided by the mean value) of angular velocity range
Speed reduction	Difference in angular velocity range between the last and the first five seconds of elbow flexion as a percentage of initial angular velocity range
Flexion number	Number of flexion/extensions during 20 seconds

Normal-paced 6MWD test was performed following the standard guidelines of the European Respiratory Society and the American Thoracic Society [11]; the total distance covered was recorded to the nearest meter.

### Statistical analysis

Correlations between UEF parameters (i.e., speed, range of motion, power, rise time, moment, speed variability, speed reduction, and flexion number), pulmonary function measures (i.e., FEV_1_, FVC, PEFR, MIP, and MEP), CAT score, and 6MWD results were assessed using linear Pearson correlations for normally distributed or the Spearman’s rank for not normally distributed samples (both reported as *r* value). Analysis of variance (ANOVA) tests were performed to assess independent associations between UEF parameters and pulmonary function measures, adjusted with age, gender, and BMI.

Furthermore, we previously developed a UEF score, which was validated based on the Fried frailty index. Of note, “frailty” is used to identify homeostenotic older adults with low physiological reserves, vulnerability to illness, and high risk of disability, institutionalization, and mortality. Frailty is a more sensitive predictor of health outcomes than is age. This score includes BMI and speed, flexibility, moment, speed variability, speed reduction and flexion number. A UEF score of 0 corresponds to extreme resilience and a score of 1 corresponds to extreme frailty. Correlation between UEF score, pulmonary function measures, CAT score, and 6MWD results were also assessed. Cut-offs of 0.01–0.19: very weak, 0.20–0.39: weak, 0.40–0.59: moderate, 0.60–0.79: strong, and 0.80–1.00: very strong were selected for correlations.

Further, a multivariable linear regression model was used to determine the association between UEF and 6MWD tests. Collinearity among UEF parameters were assessed using variance inflation factor (VIF) values [26]. VIF values larger than 10 was considered for collinearity of the parameter. In this model, independent association between UEF parameters and walking distance was assessed, considering 6MWD as the dependent variable, and UEF parameters (with significant correlation with 6MWD), age, gender, and body mass index (BMI) as independent variables. The analysis was repeated considering FEV_1_ as an additional independent variable. The goodness of fit was examined by the *r* correlation value, as well as testing for the normality of the residuals (Shapiro-Wilk *W*-test) and comparing the predicted and measured 6MWD values using paired *t*-test. Considering a cut-off of 350 m for poor versus normal 6MWD [18], sensitivity and specificity of predicting poor functional capacity using the UEF model were determined. For this purpose, a logistic regression model was used with poor vs normal functional capacity (based on 6MWD performance) as the dependent variable, and UEF and demographic parameters (with significant independent association with 6MWD) as independent variables. All analyses were conducted using JMP version 11 (SAS Institute, Inc., Cary, NC), and statistical significance was set at *p* < 0.05.

## Results

### Participants

Forty-nine participants were recruited; mean (standard deviation—SD) age, BMI, and measured FEV_1_/FVC values were 72 (8) years (ranged from 57 to 93 years), 28.4 (5.5) kg/m^2^, and 48 (15) %, respectively. All sociodemographic data, clinical information, and outcome measures are reported in Table 2.

**Table 2:** Outcome measures. For each gender the mean and standard deviation (SD) values for demographic information, subjective clinical measures, pulmonary function measures, and functional capacity measures are presented.

Variable	Male	Female	Combined
Demographic Information			
Number (% of the group)	30 (61%)	19 (39%)	49
Age, year (SD)	72 (8)	71 (6)	72 (8)
Stature, cm (SD)	174.6 (8.5)	158.8 (6.1)	168.5 (10.9)
Body mass, kg (SD)	86.5 (15.8)	71.4 (17.4)	80.7 (17.9)
BMI, kg/m^2^ (SD)	28.4 (4.8)	28.3 (6.6)	28.4 (5.5)
Subjective Clinical Outcomes			
Smoking, Pack per year (SD)	60 (18)	60 (17)	60 (17)
Smoking history, year (SD)	40 (13)	38 (15)	39 (14)
COPD severity-Total CAT score, 0–40 scale (SD)	15.37 (7.18)	17.29 (6.41)	16.11 (6.89)
Pulmonary Function Measures			
FEV_1_, Liters (SD)	1.90 (0.69)	1.22 (0.56)	1.61 (0.72)
Percent Predicted FEV_1_, percentage (SD)	0.62 (0.21)	0.57 (0.24)	0.60 (0.22)
FVC, Liters (SD)	3.87 (0.84)	2.48 (0.72)	3.28 (1.05)
Percent Predicted FVC, percentage (SD)	0.92 (0.14)	0.88 (0.21)	0.91 (0.17)
PEFR, Liter/sec (SD)	6.42 (2.16)	4.13 (1.61)	5.45 (2.24)
FEV_1_/FVC, percentage (SD)	0.49 (0.14)	0.48 (0.14)	0.49 (0.14)
MIP, cmH_2_O (SD)	84.19 (23.13)	59.00 (18.51)	72.23 (24.39)
MEP, cmH_2_O (SD)	109.15 (47.62)	85.28 (35.36)	97.84 (43.42)
Median GOLD stages (% stage 4)	2 (10%)	3 (16%)	2 (12%)
Functional Capacity Measures			
6MWD, m (SD)	406.71 (99.36)	291.19 (105.11)	356.76 (115.99)
Speed, deg/s (SD)	989.20 (378.97)	814.39 (224.29)	921.42 (335.22)
Range of motion, deg (SD)	110.74 (29.66)	117.92 (25.61)	113.53 (28.11)
Power, deg^2^/s^3^ x 100000 (SD)	168.88 (114.28)	88.58 (41.56)	137.10 (100.08)
Rise time, s/100 (SD)	29.46 (10.21)	30.50 (8.20)	29.86 (9.41)
Moment, Nm (SD)	1.66 (0.72)	0.60 (0.22)	1.24 (0.77)
Speed variability, percentage (SD)	17.58 (17.53)	18.60 (23.92)	17.98 (20.01)
Speed reduction, percentage (SD)	5.29 (13.55)	7.41 (11.39)	6.11 (12.68)
Flexion number (SD)	22.73 (6.67)	18.82 (4.39)	21.21 (6.15)

BMI: body mass index. COPD: chronic obstructive pulmonary disease. FEV_1_: forced expiratory volume in one second. FVC: forced vital capacity. PEFR: peak expiratory flow rate. MIP: maximal inspiratory pressure. MEP: maximal expiratory pressure. 6MWD: 6 minute walk distance.

### Association between UEF and 6MWD

There were significant correlations between UEF speed, power, moment, speed variability, flexion number, and UEF score with 6MWD (Table 3). Among these, speed, power, moment, flexion number, and UEF score revealed moderate to strong (*r* = 0.44–0.62), and speed variability (*r* = -0.36) showed weak correlations with 6MWD. Among all UEF parameters, elbow moment demonstrated the strongest correlation with 6MWD (*r* = 0.62, *p* < 0.001).

**Table 3:** Association between UEF parameters, 6MWD, and pulmonary function measures. Pearson correlation coefficients (*r*) are presented. P-values for independent associations between UEF parameters and pulmonary function measures (adjusted with age, gender, and BMI) are presented in Parenthesis.

Functional Capacity	6MWD	FEV_1_	FVC	PEFR	MIP	MEP
Speed	*p* < 0.01* (*p* = 0.02*) *r* = 0.50	*p* = 0.14 (*p* = 0.97) *r* = 0.22	*p* = 0.04* (*p* = 0.53) *r* = 0.30	*p* = 0.18 (*p* = 0.80) *r* = 0.21	*p* = 0.01* (*p* = 0.13) *r* = 0.40	*p* = 0.21 (*p* = 0.61) *r* = 0.20
Range of motion	*p* = 0.23 (*p* = 0.05) *r* = 0.20	*p* = 0.56 (*p* = 0.39) *r* = 0.08	*p* = 0.78 (*p* = 0.97) *r* = 0.04	*p* = 0.49 (*p* = 0.38) *r* = 0.10	*p* = 0.44 (*p* = 0.38) *r* = 0.14	*p* = 0.13 (*p* = 0.83) *r* = 0.26
Power	*p* < 0.01* (*p* = 0.42) *r* = 0.44	*p* = 0.05 (*p* = 0.97) *r* = 0.30	*p* < 0.01* (*p* = 0.32) *r* = 0.38	*p* = 0.04* (*p* = 0.84) *r* = 0.31	*p* < 0.001* (*p* = 0.04*) *r* = 0.52	*p* = 0.23 (*p* = 0.95) *r* = 0.20
Rise time	*p* = 0.33 (*p* = 0.25) *r* = 0.-17	*p* = 0.99 (*p* = 0.85) *r* = 0.00	*p* = 0.98 (*p* = 0.80) *r* = 0.00	*p* = 0.83 (*p* = 0.98) *r* = -0.31	*p* < 0.01* (*p* = 0.12) *r* = -0.42	*p* = 0.06 (*p* = 0.41) *r* = -0.32
Moment	*p* < 0.0001* (*p* = 0.05*) *r* = 0.62	*p* < 0.0001* (*p* = 0.16) *r* = 0.55	*p* < 0.0001* (*p* = 0.51) *r* = 0.64	*p* < 0.0001* (*p* = 0.22) *r* = 0.55	*p* < 0.0001* (*p* < 0.01*) *r* = 0.69	*p* = 0.04* (*p* = 0.72) *r* = 0.33
Speed variability	*p* = 0.03* (*p* = 0.11) *r* = -0.36	*p* = 0.78 (*p* = 0.97) *r* = -0.04	*p* = 0.10 (*p* = 0.69) *r* = -0.24	*p* < 0.01* (*p* = 0.85) *r* = -0.42	*p* = 0.33 (*p* = 0.87) *r* = -0.17	*p* = 0.29 (*p* = 0.31) *r* = -0.17
Speed reduction	*p* = 0.24 (*p* = 0.70) *r* = -0.20	*p* = 0.04* (*p* = 0.07) *r* = -0.32	*p* = 0.42 (*p* = 0.80) *r* = -0.12	*p* = 0.09 (*p* = 0.18) *r* = -0.25	*p* = 0.61 (*p* = 0.96) *r* = -0.10	*p* < 0.01* (*p* = 0.02*) *r* = -0.42
Flexion number	*p* < 0.01* (*p* = 0.04*) *r* = 0.50	*p* = 0.77 (*p* = 0.83) *r* = 0.04	*p* = 0.27 (*p* = 0.90) *r* = 0.17	*p* = 0.73 (*p* = 0.54) *r* = 0.05	*p* = 0.08 (*p* = 0.14) *r* = 0.28	*p* < 0.01* (*p* = 0.29) *r* = 0.42
UEF score	*p* = 0.002* (*p* = 0.01*) *r* = 0.51	*p* = 0.42 (*p* = 0.95) *r* = 0.13	*p* = 0.31 (*p* = 0.73) *r* = 0.16	*p* = 0.70 (*p* = 0.65) *r* = 0.06	*p* = 0.04* (*p* = 0.15) *r* = 0.35	*p* = 0.05 (*p* = 0.15) *r* = 0.32
6MWD	-	*p* < 0.0001* (*p* < 0.01*) *r* = 0.69	*p* < 0.0001* (*p* = 0.03*) *r* = 0.63	*p* < 0.0001* (*p* < 0.01*) *r* = 0.67	*p* = 0.02* (*p* = 0.50) *r* = 0.41	*p* < 0.01* (*p* = 0.05) *r* = 0.51

6MWD: 6-minute walk distance. FEV_1_: forced expiratory volume in one second. FVC: forced vital capacity. PEFR: peak expiratory flow rate. MIP: maximal inspiratory pressure. MEP: maximal expiratory pressure.

*: The asterisk symbol represents a significant correlation.

**Fig 2:**
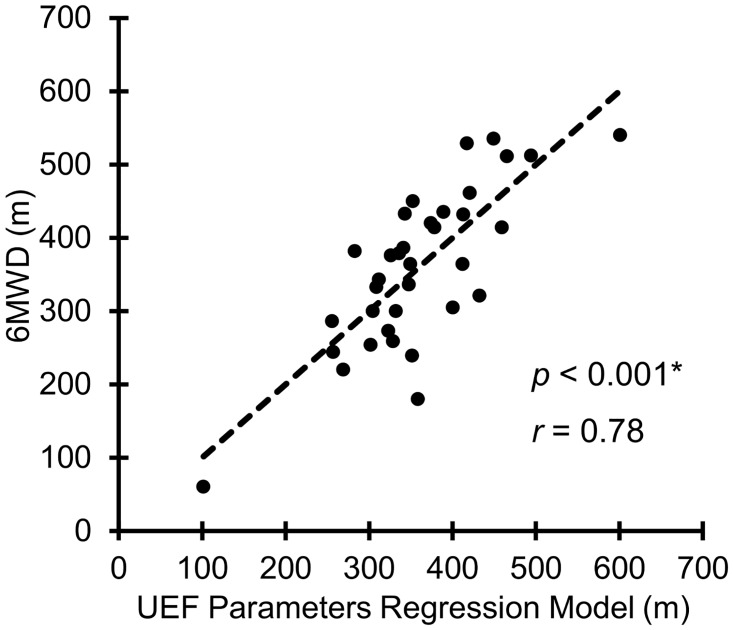
Multivariable regression model; dependent variable: 6MWD and independent variables: UFM parameters with significant correlation with 6MWD (i.e., speed, power, moment, speed variability, flexion number), age, gender, and BMI.

Results from the multivariable regression model revealed that speed, power, and moment UEF parameters are independent predictors of 6MWD (Table 4); a correlation of 0.78 (*p* < 0.001) was determined between the multivariable regression model and 6MWD within the current sample using UEF and demographic parameters presented in Table 4 (Fig 2); the correlation improved to 0.87 when FEV_1_ was added to the model as an independent variable. A correlation of 0.69 (*p* < 0.0001) was achieved in predicting 6MWD, when only speed, power, and moment UEF parameters, without demographic parameters, were used as independent variables; the correlation improved to 0.84 when FEV1 was added to the model as an independent variable. Of note, three UEF parameters (i.e., speed, power, and moment) were still independent predictors of 6MWD even after adding FEV_1_ as an additional variable. Among UEF parameters, only power was collinear with remaining UEF parameters (VIF = 12.5). However, power was kept in the model, since the purpose of this model was to provide best prediction of 6MWD using UEF, rather than investigating specific association between each UEF parameter with 6MWD. Age, gender, and BMI were not significantly associated with 6MWD when considered as independent variables in addition to UEF parameters in the model. Using the UEF model prediction based on speed, power, and moment, paired *t*-test results showed no significant difference between measured and predicted 6MWD values (*t* = 2.13, *p* = 0.34), and residuals were normally distributed (*W* = 0.95, *p* = 0.57). Moreover, considering a cut-off of 350 m the model predicted poor 6MWD with a sensitivity and specificity of 0.75 and 0.89, respectively (receiver operating characteristic (ROC) curve = 0.86). Of note, in this model only speed, power, and moment UEF parameters were used, as they showed significant independent association with 6MWD.

**Table 4:** Results of the multivariable regression model. Dependent variable: 6MWD; independent variables: UEF parameters (with significant linear correlation with 6MWD), age, gender, and, BMI.

Independent Variables	Parameter Estimates	Standard Errors	*t*-Ratio	*p*-value	95% CI (Lower)	95% CI (Upper)
Speed	0.28	0.14	2.04	0.05*	0.00	0.56
Power	-1.15x10^-05^	4.11x10^-06^	-2.80	0.01*	-2.00x10^-05^	-3.06x10^-06^
Moment	137.18	47.15	2.91	0.01*	40.27	234.10
Speed variability	-0.95	0.86	-1.11	0.28	-2.72	0.81
Flexion Number	-0.29	3.19	-0.09	0.93	-6.85	6.27
Gender	-2.34	23.58	-0.10	0.92	-50.81	46.12
Age	-1.01	2.01	-0.50	0.62	-5.13	3.12
BMI	-3.31	2.81	-1.18	0.25	-9.08	2.47

*: The asterisk symbol represents a significant independent association.

### Association between UEF and pulmonary function

**Fig 3:**
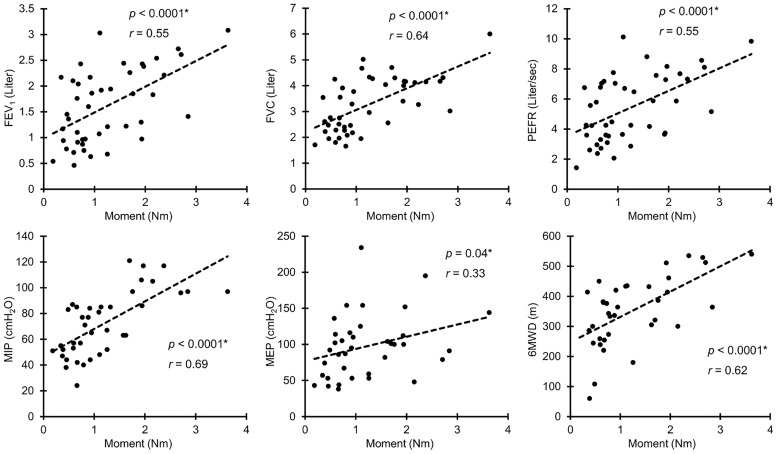
Elbow moment, overall, demonstrated the strongest association with pulmonary function measures and 6MWD among UEF parameters. The asterisk symbol represents a significant correlation.

Among UEF parameters, only elbow moment showed significant correlations with all pulmonary function measures (*r* = 0.35–0.69, *p* < 0.02); except for the MEP measure, elbow moment was moderately to strongly correlated with pulmonary function measures (Table 3 and Fig 3). Overall results demonstrated that comparable associations exist between elbow moment and 6MWD with pulmonary function measures. Other than elbow moment, weak to moderate correlations were determined between UEF speed and power parameters with FVC and MIP measures (*r* = 0.30–0.52, *p* < 0.04, Table 3). No significant correlation was observed between any of the UEF parameters (or the 6MWD) and CAT score (*r* < 0.28, *p* > 0.08).

## Discussion

### Upper-extremity motion and walking

As hypothesized, UEF parameters showed significant correlations with 6MWD. The strongest correlation was observed between elbow moment and 6MWD. Elbow moment, as defined in Table 1, represents the maximum moment imposed on elbow while performing the repetitive elbow flexion task. This parameter was measured by estimating the angular acceleration and angular velocity from the sensors, and moment of inertia of upper-extremity from the anthropometry data. Therefore, UEF moment is considered as a measure of muscle strength (dynapenia) in providing a fast movement. Interestingly, elbow moment as measured within our previous work (15), showed the strongest correlation among all UEF parameters with the grip strength test (*r* = 0.51–0.67, *p* < 0.0001). Previous work has demonstrated associations between upper-extremity muscle strength (measured using grip strength test) with lower-extremity muscle strength and 6MWD in COPD patients [27]. These association suggest that assessment of lower and upper-extremity strength may both provide a measure of functional capacity among COPD patients.

Furthermore, moderate correlations between speed and power among UEF parameters with 6MWD were observed. Elbow speed and power parameters represent how fast and with what acceleration participants moved their upper-extremities. Overall, in addition to muscle strength, these observations suggest that lack of speed, as a determinant factor of functional capacity in COPD, could be identified using the UEF test. Associations between speed of movement and 6MWD has been reported previously. For instance, walking speed, measured by a 30-meter walk test, especially fast walking, was identified as a determinant factor of functional capacity in COPD patients [28]. These findings, therefore, showed a potential advantage of the UEF test that covers slowness in limb movement as a marker of muscle function [27] beyond those of an isometric muscle strength tests such as the hand grip strength assessment.

### Upper-extremity motion and pulmonary function

As reported in Table 3, several UEF parameters, specifically elbow moment, speed, and power of movement, are closely associated with pulmonary function measures. This is in agreement with previously observed correlation between upper-extremity strength (measured by grip strength) and pulmonary function [21,29]. As previously investigated, compromised pulmonary function is correlated not only with low respiratory muscle performance (e.g., measured by MIP) but also with peripheral muscle weakness [21,27]. This association suggests that functional capacity may be related to lack of oxygen transport and ventilatory limitation, as well as fatigue and lack of strength in peripheral muscles [30]. In addition to nutritional factors that can lead to muscle mass loss in COPD patients, systematic reduction in muscle strength and function is also evident in this population, which can result from hypoxemia, hypercapnia, and steroid treatments, as well as inactivity and muscle deconditioning [20,21,31]. In agreement with previous research, we also observed a close correlation between elbow moment, which is representative of upper-extremity muscle strength, with pulmonary function measures (FEV_1_, FVC, and PEFR), as well as MIP and MEP values (Table 3 and Fig 3).

In spite of UEF parameters that are related to speed and strength of upper-extremity movements, flexion number, flexibility, and fatigue related UEF parameters (i.e., range of motion, speed reduction, and speed variability) demonstrated weaker correlations with pulmonary function measures (Table 3). Impaired muscle oxidative capacity has been suggested as the reason for excessive fatigue in lower-extremity exercise in COPD patients [32]. Therefore, although weak to moderate correlations were observed between UEF fatigue-related parameters and FEV_1_, PEFR, and MEP values, the short 20-second UEF test may not be long enough to cause excessive fatigue in COPD patients.

### Limitations

As with measurement limitations for gait-based measures, upper-extremity disability or injury may limit UEF measurement. Further, due to small sample size, current results should be considered preliminary and need further validation among larger samples. Also, a small percentage of participants were at severe COPD stage (overall 12% were at stage 4 based on GOLD standard), and therefore, the current sample may not very adequately represent those with functional/gait impairments. Also, the current study lacks intra- and inter-rater reliability assessments; however, as we previously validated the UEF test among a larger sample of older adults within different experimental settings, we have reported significant weak to strong correlations between all UEF parameters and gait speed (*r* = 0.38–0.68; *p* < 0.001) (15). Lastly, although strong correlation was observed here between UEF and 6MWD tests, the association between UEF outcomes and long-term prospective clinical measures, including risk of exacerbations, hospitalization, and mortality should be assessed.

### Conclusions

We evaluated the UEF test as a functional capacity assessment tool in older adults with COPD, which shows promise for providing an additional test for those who are unable to walk. The UEF test demonstrated that speed and strength of upper-extremity motion were closely associated with pulmonary function measures and the 6MWD test. Although previous work suggested that strength and function of upper-extremity muscles may be better preserved than that of lower-extremity muscles [31], current results provide evidence that upper-extremity motion assessment may be useful in assessing systematic muscle dysfunctioning in COPD patients. However, it should be acknowledged that there are differences in 6MWD and UEF tests, since walking is inherently a more complicated and burdensome task, and therefore, UEF should not be considered as a replacement for 6MWD but an alternative measure for those who are unable to perform the gait test. Assessment of functional capacity is critical in directing COPD care and in predicting COPD health outcomes. Therefore, further studies with different experimental settings are required to validate the efficacy of functional capacity assessment using upper-extremity motor performance in older adults with COPD.

## Supporting information

S1 Table Sociodemographic information, subjective clinical outcomes, pulmonary function measures, and functional capacity measures. (XLSX)
